# An estradiol-independent BDNF-NPY cascade is involved in the antidepressant effect of mechanical acupuncture instruments in ovariectomized rats

**DOI:** 10.1038/s41598-018-23824-2

**Published:** 2018-04-11

**Authors:** Su Yeon Seo, Ji-Young Moon, Suk-Yun Kang, O. Sang Kwon, Sunoh Kwon, Se kyun Bang, Soo Phil Kim, Kwang-Ho Choi, Yeonhee Ryu

**Affiliations:** 1 Korea Institute of Oriental Medicine 1672 Yuseongdae-ro, Yuseong-gu, Daejeon 34054 Republic of Korea; 2 Animal and Plant Quarantine Agency 177, Hyeoksin 8-ro, Gimcheon-si, Gyeongsangbuk-do Republic of Korea

## Abstract

Menopause-related depression devastates women’s quality of life after middle age. Previous research has shown that estrogen hormone therapy has serious adverse effects; thus, complementary and integrative therapies have been considered clinically. The present study investigates whether stimulation of an acupoint using a mechanical acupuncture instrument (MAI) can mitigate depression-like behavior caused by estrogen deficiency in ovariectomized (OVX) rats. The animals were divided into Sham OVX, OVX, OVX + Sameumgyo (SP6) and OVX + NonAcu (non-acupuncture point) groups. MAI stimulation significantly increased the total distance traveled in the open-field test and the number of open-arm entries in the elevated plus maze and decreased the duration of immobility in the forced swim test. In addition to this decrease in depression-like behavior, brain-derived neurotrophic factor (BDNF) and neuropeptide Y (NPY) release increased in the hippocampus in response to MAI treatment, but estradiol levels did not recover. Furthermore, microinjection of the BDNF receptor antagonist ANA-12 (0.1 pmol/1 μl) into the hippocampus before MAI stimulation significantly suppressed the recovery of NPY levels. Taken together, these findings indicate that MAI stimulation at SP6 facilitates an estradiol-independent BDNF-NPY cascade, which may contribute to its antidepressant effects in OVX rats, an animal model of menopausal disorders.

## Introduction

Depression is a significant public health problem due to its high prevalence and devastating impact on both individuals and society. The ovaries produce less estrogen and progesterone over time until they shut down completely. A reduction in the levels of these hormones triggers the effects of menopause. Many older women suffer from menopause-related symptoms, one of which is depression^1^. Estrogen replacement therapy has been applied to treat menopause-related symptoms, including depression; however, it also results in some severe adverse effects, including thrombotic events, breast cancer and dementia^2^. Acupuncture is considered an alternative treatment for menopause-related symptoms, which is supported by a meta-analysis of randomized controlled trials that used acupuncture to improve menopause-related symptoms^3^. However, few preclinical studies have explored how acupuncture improves menopause-related symptoms, despite the clinical validity of acupuncture.

The hippocampus plays an important role in depression^4^. The hippocampus is adjacent to the edge of the cerebral cortex of the medial temporal lobe. These regions have functional roles in memory and mood regulation. The hippocampal volume is related to declarative, episodic, and contextual learning and memory and various mood disorders such as depression, anxiety and bipolar disorder^5^. Hippocampal atrophy is observed in depression patients and can be improved by antidepressant treatments^6,7^. Antidepressant treatment can increase brain-derived neurotrophic factor (BDNF) synthesis and signaling in the hippocampus and prefrontal cortex^8–10^. The increased BDNF and phosphorylated TrkB can activate the extracellular signal-regulated kinase (ERK)-cAMP response element binding protein (CREB) signaling pathway in the hippocampus^11–13^.

Estrogen plays an important role in cell proliferation and neurogenesis in the hippocampus^1,14^. There are three forms of estrogen: estradiol (E2), estriol and estrone^15,16^. In female rats, the density of dendritic spines or synapses on hippocampal pyramidal cells depends upon the ovarian steroid E2^17^. E2, one of the major nerve steroids in the hippocampus, interacts with various neurotrophins to regulate hippocampal function^18,19^. Ovariectomized (OVX) animal models of estrogen deficiency have symptoms similar to those of women with menopause-related depression, such as increased body weight, bone loss and decreased estrogen receptor levels^20,21^. The physiological functions of estrogenic compounds are modulated largely by estrogen receptor subtypes alpha (ERα) and beta (ERβ). In rodents, ERβ plays a greater role in anxiety-like behavior and depression than ERα^22–25^. ERβ is known to be important for the modulation of affective behavior by E2. Previously, subcutaneous administration of selective estrogen receptor modulators that have higher affinity for ERβ than for ERα was shown to produce anti-anxiety and anti-depressant-like effects in OVX rats in several tasks^26^. Furthermore, permanent knockout of ERβ increases anxiety and depression behavior in mice^6,22,27^.

BDNF, a secretory protein in the neurotrophin family, plays a crucial role in numerous aspects of brain development and function, including neurogenesis^28–30^. Thus, control of hippocampal BDNF is an important target for the treatment of depression. According to recent research, E2 and BDNF control neurodevelopment and neural plasticity^31^. Direct interaction between these factors induces BDNF upregulation, promotes neuronal growth, and preserves neuronal plasticity^31^. These results suggest that estrogen can regulate BDNF release.

Increased BDNF levels can facilitate expression of neuropeptides such as neuropeptide Y (NPY) in the hippocampal neurons^32,33^. In the hippocampus, NPY has robust effects, including neuroprotection and synaptic transmission, that play important roles in the pathology of several depression- and anxiety-related disorders^34,35^. Recent studies show that administration of exogenous E2 or BDNF increases NPY in the hippocampus^36,37^. Therefore, increased levels of BDNF and, subsequently, NPY could relieve estrogen-deficiency-related depressive symptoms. Thus, antidepressants are thought to act by activating an E2-BDNF-NPY cascade in the hippocampus. However, most studies of depression focus on the serotonin system.

In acupuncture research, stimulation of acupuncture points has been shown to regulate E2, BDNF and NPY^38–41^. Despite various previous studies of the association among E2, BDNF and NPY, research into how acupuncture modulates this system is generally lacking. The present study applied the Sanyinjiao (SP6) point. Acupuncture stimulation at the SP6 point has been shown to be effective in improving women’s general health. Recent studies indicate that acupuncture at SP6 is effective in increasing estradiol and in significantly reducing follicle stimulating hormone (FSH). Hormonal changes caused by SP6 stimulation also lead to reduced hot flashes associated with premenopausal women^42–45^. The aim of this study was to investigate the pathway mediating the antidepressant effect of mechanical acupuncture instrument (MAI) stimulation of SP6 using an estrogen-deficient rat model of depression. In detail, the antidepressant effect of SP6 stimulation was examined in relation to the E2-BDNF-NPY pathway in estrogen deficiency depression in OVX rats.

## Results

### MAI stimulation of the SP6 acupuncture point significantly mitigated depression-like behavior in OVX rats

**Figure 1:**
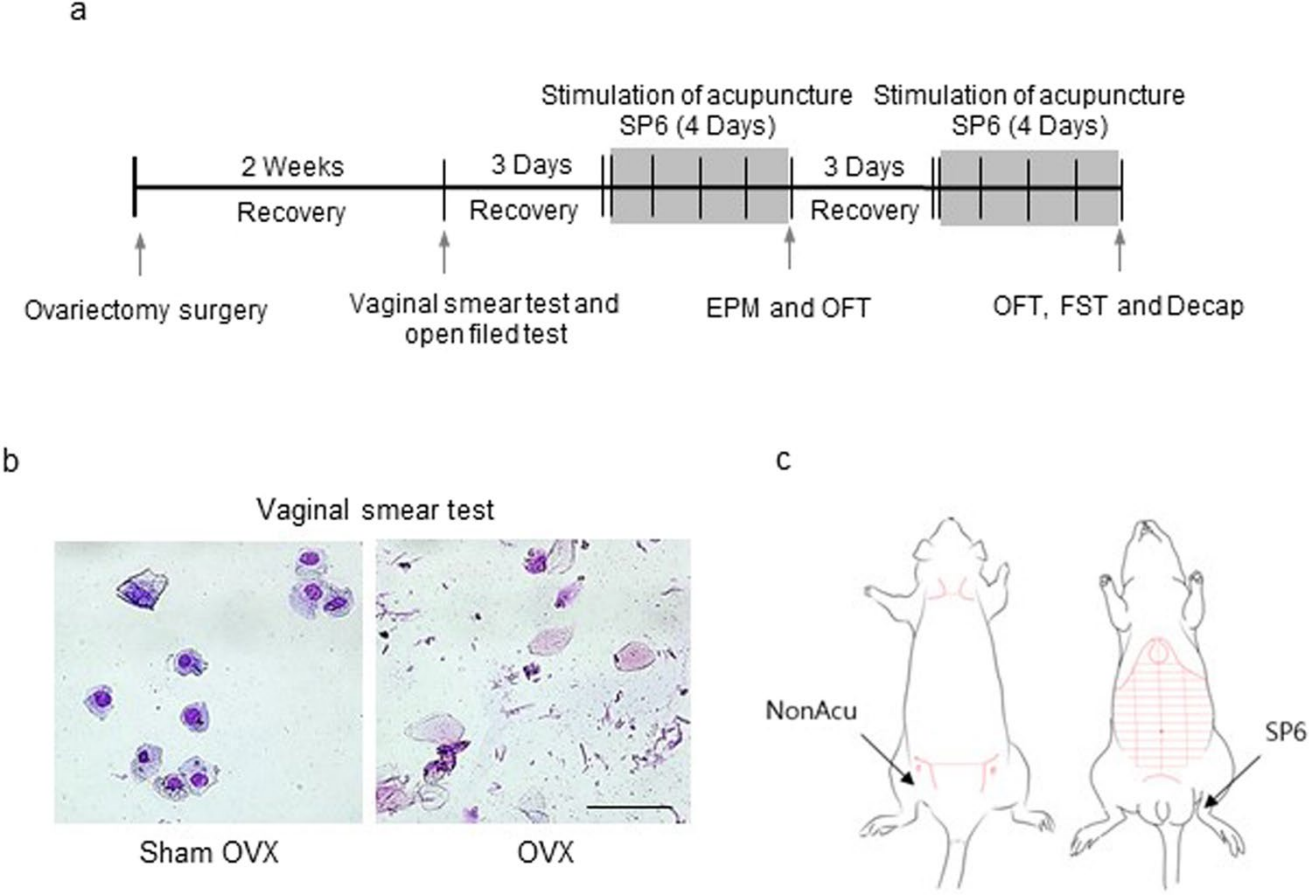
Schematic diagram and map of acupuncture point SP6. (**a**) Schematic diagram showing the stimulation of acupuncture of the rats. (**b**) The stages of the estrous cycle as characterized by vaginal cytology in Sham OVX and OVX rats. (**c**) Acupuncture stimulation was performed at acupuncture point Sanyinjiao (SP6) and a non-acupuncture point location (upper part of the left buttock).

**Figure 2:**
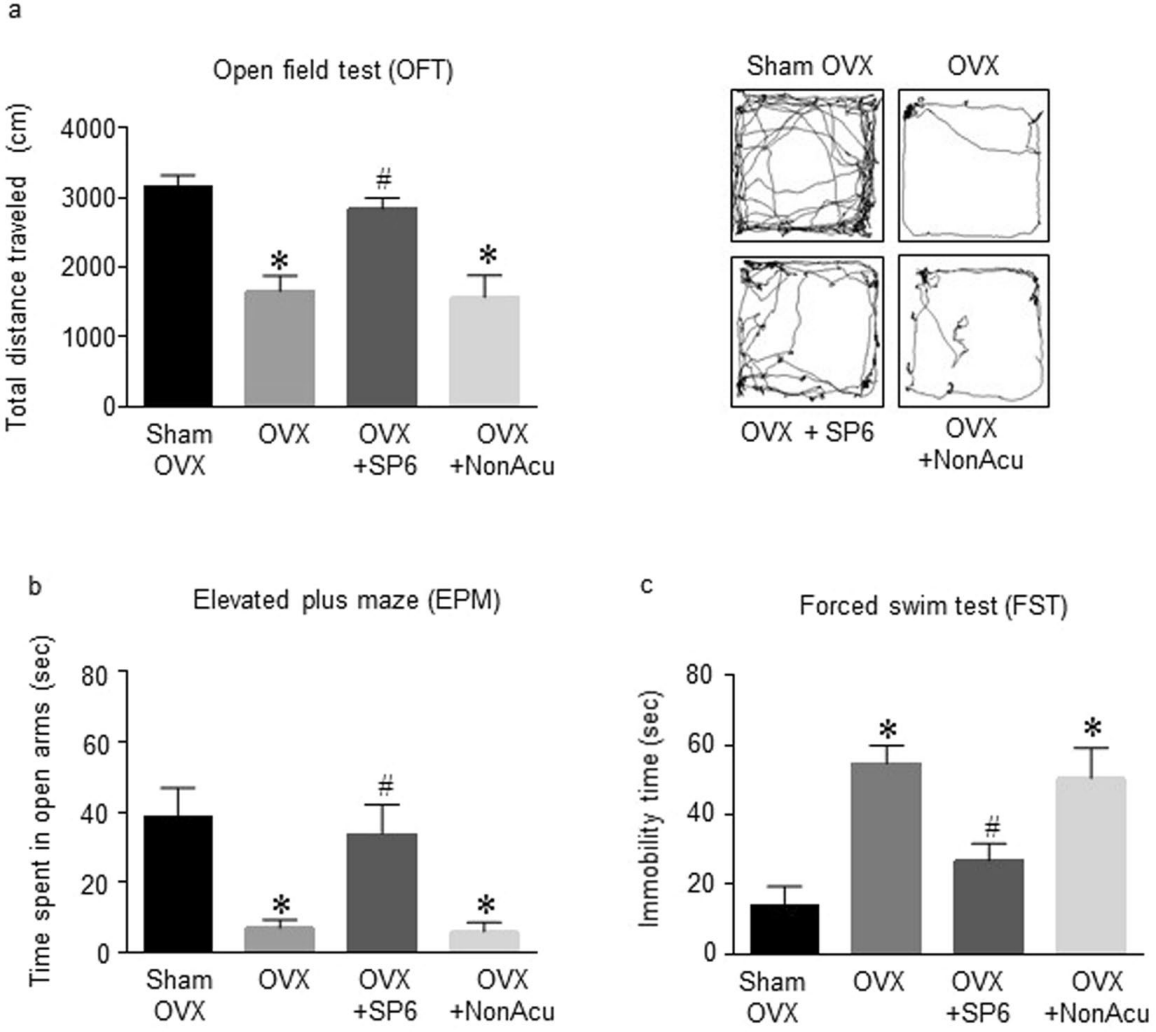
Effects of acupuncture stimulation on depression-like behaviors. Quantification of (**a**) total distance traveled in the OFT (n = 10 for each group), (**b**) the entries into the open arms in the EPM (n = 7~8 for each group) and (**c**) immobility time during the FST (n = 7~8 for each group). The data were analyzed using repeated measures ANOVA followed by Tukey’s test. *p < 0.05 vs. Sham group; ^#^*p* < 0.05 vs. OVX group. Values are expressed as the means ± SEM.

This study was performed to determine whether acupuncture stimulation changes depression-like behavior in OVX rats. The OVX rats were treated with MAI for 4 days, and their behavior was measured 20 min after the last MAI stimulation (Fig. 1a). In the open-field test (OFT), OVX + SP6 rats traveled a significantly greater total distance than the OVX rats (F_(3,35)_ = 14.15, *df* = 38, n = 7~8 per group) but not the OVX + NonAcu (non-acupuncture point) rats (Fig. 2a). There were no significant differences among other indexes such as resting time and rearing duration (Sup. 1). On the elevated plus maze (EPM), it was shown that in the rats in the OVX + SP6 group spent significantly more time in the open arms than the OVX rats (F_(3,19)_ = 8.506, *df* = 22, n = 7~8 per group), but there was no significant difference in time spent in the closed arms or the center and center zone (Sup. 1) (Fig. 2b). In the forced swim test (FST), the immobility time in the OVX + SP6 group was lower than that of the OVX group (F_(3,19)_ = 9.659, *df* = 22, n = 7 ~ 8 per group) (Fig. 2c). These results show that stimulation with an MAI at SP6 mitigated depression-like behavior in OVX rats.

### MAI stimulation of the SP6 acupuncture point did not alter estradiol release or estrogen receptor expression in OVX rats

**Figure 3:**
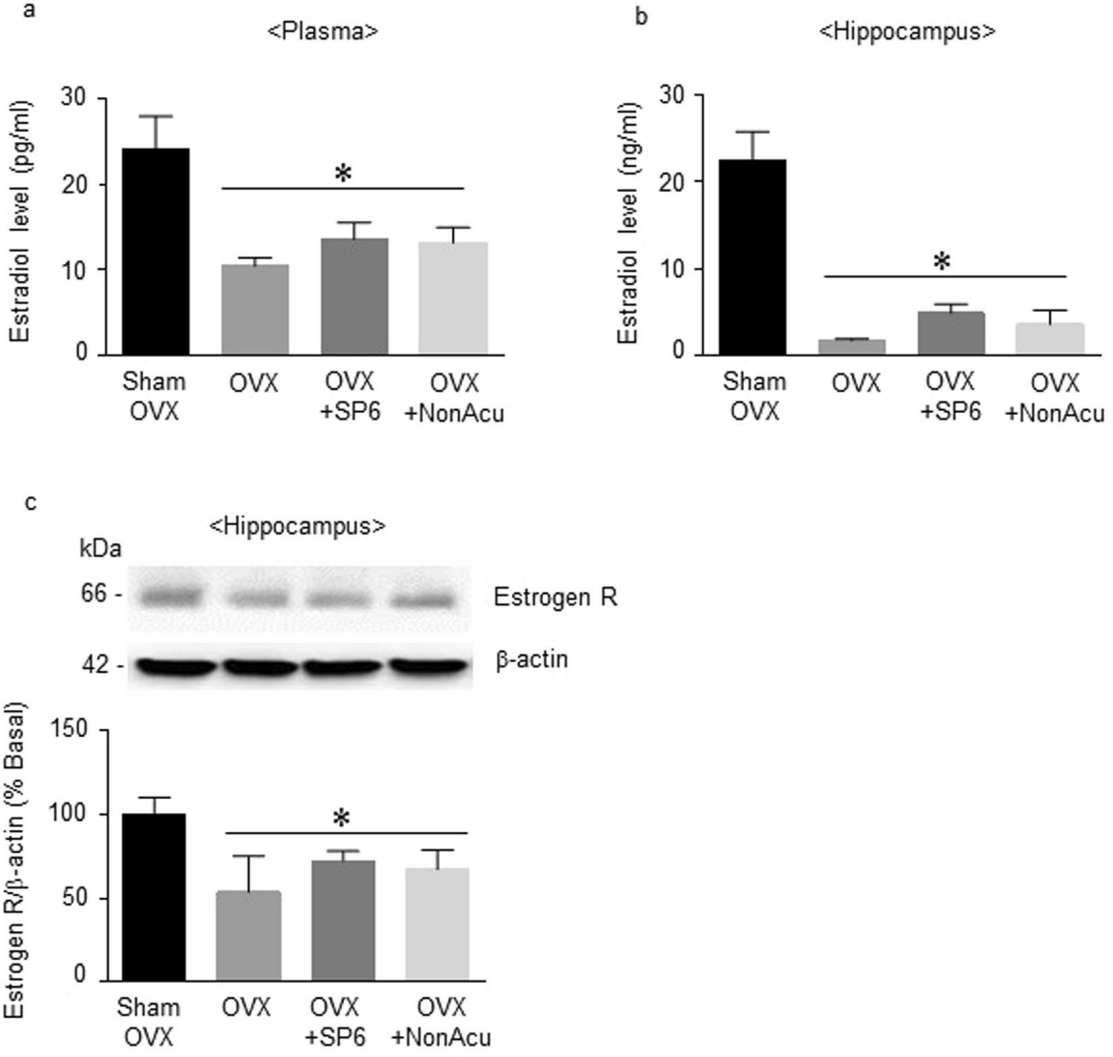
Effects of acupuncture stimulation on estradiol levels and estrogen receptor expression in the plasma and hippocampus. (**a**) The release of E2 in the plasma (n = 4 for each group). (**b**) The release of E2 in the hippocampus (n = 4 for each group). (**c**) The expression of the estrogen receptor in the hippocampus (n = 4 for each group). The data were analyzed using repeated measures ANOVA followed by Tukey’s test. *p < 0.05 vs. Sham group. Values are expressed as the means ± SEM.

Since stimulation of SP6 in the OVX rats altered depression-like behaviors, the next experiment was conducted to determine whether MAI stimulation at SP6 could increase E2 levels and estrogen receptor expression in the plasma and hippocampus. Plasma E2 levels in the OVX group were significantly lower than those of the Sham OVX rats (F_(3, 11)_ = 6.173, *df* = 14, n = 4 per group). However, the rats in the MAI stimulation group were not significantly different from the OVX and NonAcu groups (Fig. 3a). In the hippocampus, the E2 levels in the OVX rats were also decreased in comparison to the Sham OVX rats (F_(3, 11)_ = 23.66, *df* = 14, n = 4 per group), but the OVX + SP6 and OVX + NonAcu groups showed no significant difference from the OVX group in E2 levels (Fig. 3b). The estrogen receptor expression in the hippocampus was significantly decreased in the OVX rats in comparison to the Sham OVX rats (F_(3, 10)_ = 5.030, *df* = 13, n = 4 per group), but OVX did not differ significantly from the OVX + SP6 and OVX + NonAcu groups (Fig. 3c) (Sup. 2). These results show that MAI stimulation of an acupuncture point did not directly increase E2 levels or estrogen receptor expression in the OVX rats.

### MAI stimulation of the SP6 acupuncture point significantly increased the levels of hippocampal BDNF and phosphorylated TrkB receptor in OVX rats

**Figure 4:**
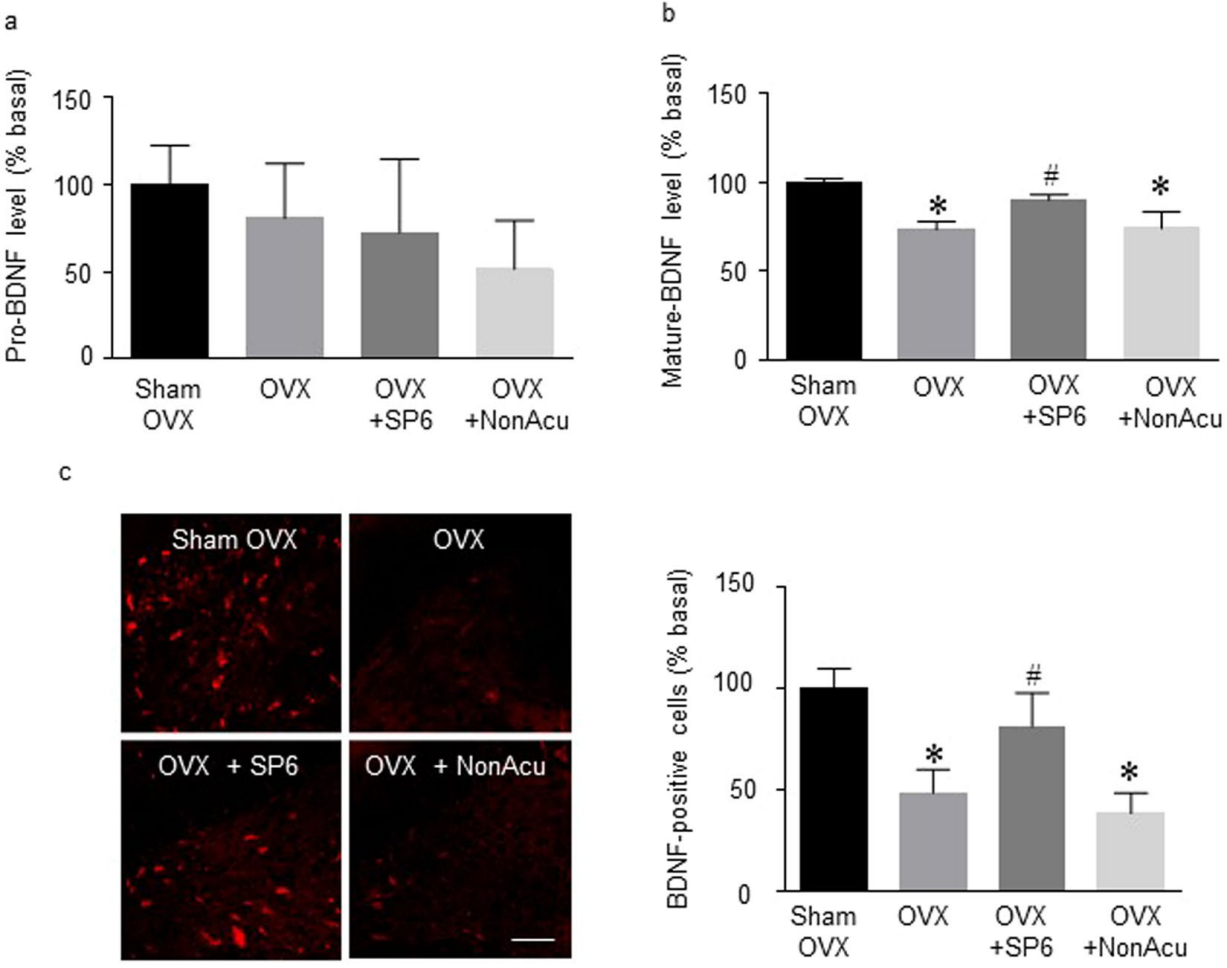
Effects of acupuncture stimulation on BDNF levels in the hippocampus. (**a**) The release of pro-BDNF in the hippocampus (n = 4 for each group). (**b**) The release of mature BDNF in the hippocampus (n = 4 for each group). (**c**) Representative micrographs showing the expression of BDNF in the hippocampus. The scale bar represents 100 μm. The results are presented as the number of BDNF-positive immunoreactive cells (n = 4~6 for each group). The mean BDNF levels are expressed as a percentage of the control. The data were analyzed using repeated measures ANOVA followed by Tukey’s test. *p < 0.05 vs. Sham group; ^#^*p* < 0.05 vs. OVX group. Values are expressed as the means ± SEM.

**Figure 5:**
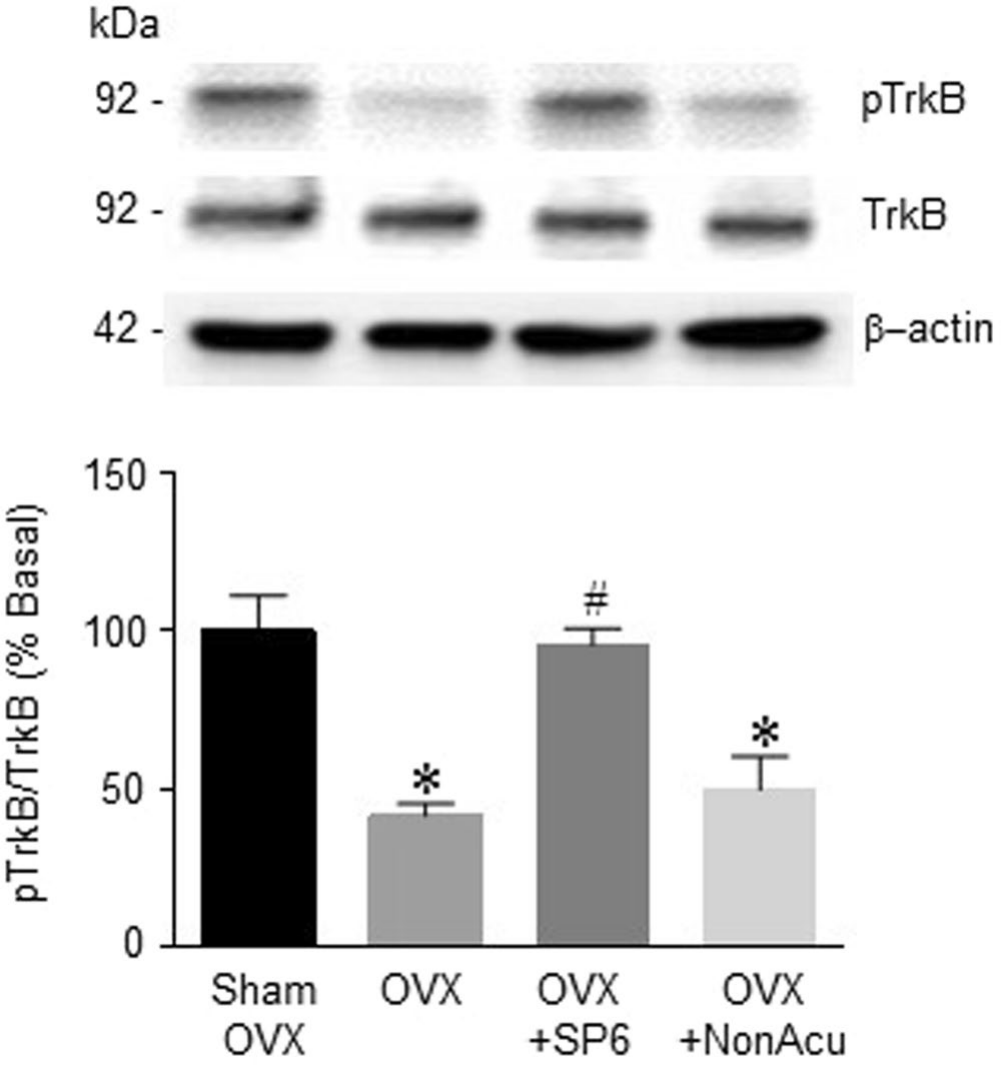
Effects of acupuncture stimulation on BDNF receptor expression in the hippocampus. The expression of phosphorylated TrkB in the hippocampus (n = 3~4 for each group). The data were analyzed using repeated measures ANOVA followed by Tukey’s test. *p < 0.05 vs. Sham group; ^#^*p* < 0.05 vs. OVX group. Values are expressed as the means ± SEM.

The next experiment determined the effect of acupuncture on the BDNF level in the hippocampus of OVX rats. The levels of pro-BDNF showed no significant difference among groups (Fig. 4a). However, the levels of mature BDNF were significantly decreased in the OVX rats compared with those in the Sham OVX rat group. The MAI stimulation at SP6 group showed significantly increased mature BDNF compared with that of the OVX rat groups (F_(3, 9)_ = 19.21, *df* = 12, n = 4 per group) (Fig. 4b). To identify changes in the BDNF level specifically within the hippocampal region, the next experiment employed an immunofluorescence assay. Immunofluorescence results showed that BDNF levels in the hippocampal CA1 region were increased in the OVX + SP6 rat group compared with those in the OVX rat group (F_(3, 11)_ = 14.91, *df* = 14, n = 4~5 per group). TrkB has the highest binding affinity for BDNF. Figure 5 shows that the OVX rat group had significantly lower phosphorylated TrkB levels than the OVX + Sham group. In addition, the OVX + SP6 rats showed significantly increased phosphorylation of TrkB compared with the OVX rat groups (F_(3, 8)_ = 9.977, *df* = 11, n = 4 per group) (Sup. 3). These results show that MAI stimulation of the SP6 acupuncture point increased the levels of mature BDNF and phosphorylated TrkB receptor in the hippocampal CA1.

### MAI stimulation of an acupuncture point significantly increased the level of hippocampal NPY in OVX rats

**Figure 6:**
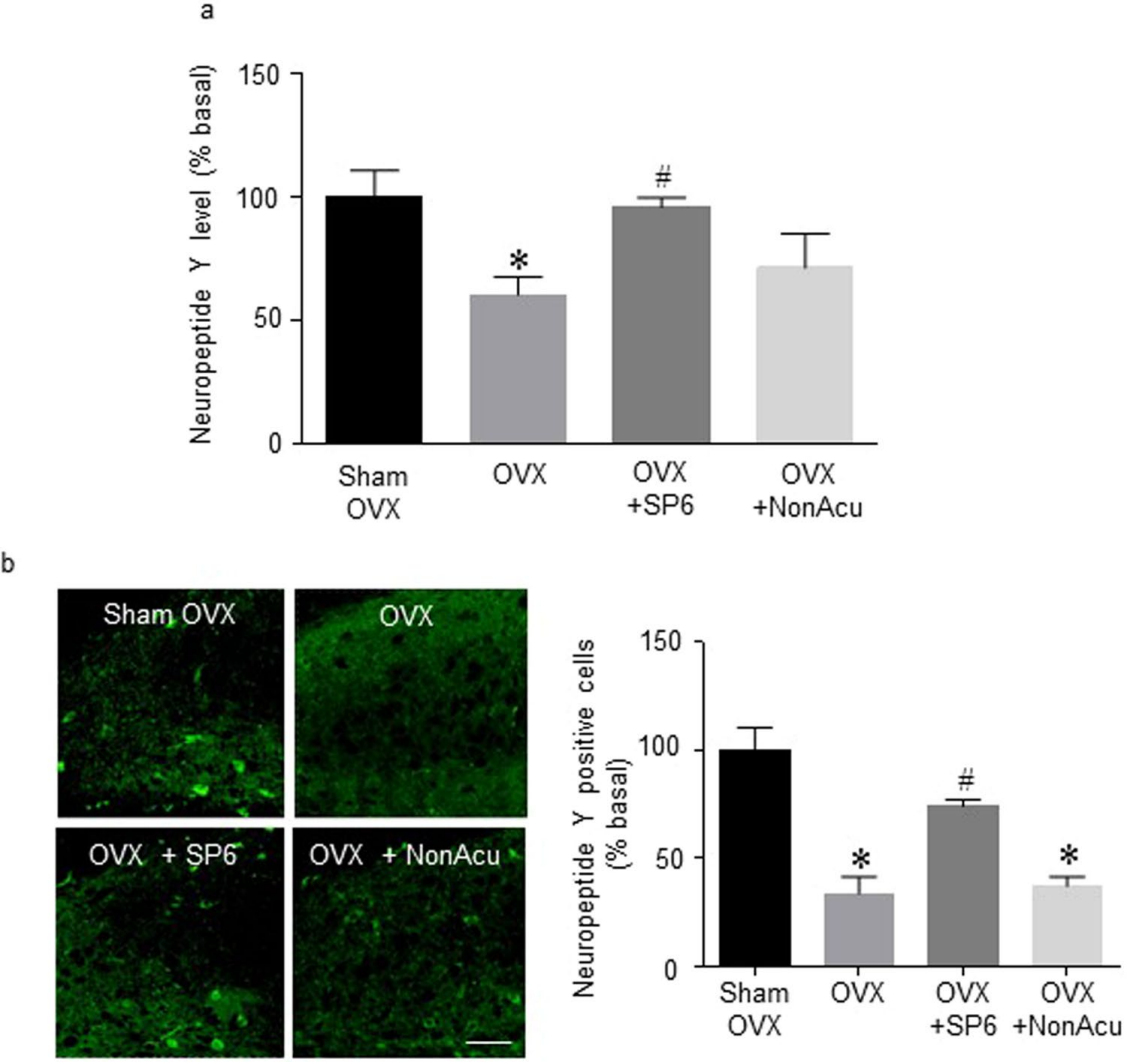
Effects of acupuncture stimulation on NPY levels in the hippocampus. (**a**) The release of NPY in the hippocampus (n = 4 for each group). (**b**) Representative micrographs showing the expression of NPY expression in the hippocampus. The scale bar represents 100 μm. The results are presented as the total number of NPY-immunoreactive cells (n = 4~6 for each group). The mean NPY levels are expressed as a percentage of the control. The data were analyzed using repeated measures ANOVA followed by Tukey’s test. *p < 0.05 vs. Sham group; ^#^*p* < 0.05 vs. OVX group. Values are expressed as the means ± SEM.

This experiment was conducted to determine whether MAI stimulation of SP6 regulates NPY release in the hippocampus of OVX rats. The level of NPY in the hippocampus significantly decreased in the OVX rats compared with that in the Sham OVX rats. Compared with this decreased NPY level in OVX rats, the OVX + SP6 rat group showed a significant increase (F_(3,11)_ = 3.946, *df* = 14, n = 4~5 per group) (Fig. 6a). Immunofluorescence results showed that NPY release increased in the OVX + SP6 rat group compared with that in the OVX rat group in the hippocampus CA1 (F_(3,15)_ = 20.09, *df* = 18, n = 4~5 per group) (Fig. 6b). These results show that MAI stimulation of the SP6 acupuncture point also altered NPY levels in the hippocampal CA1.

### NPY levels increased as a result of the increase in BDNF in response to MAI stimulation of the SP6 acupuncture point

**Figure 7:**
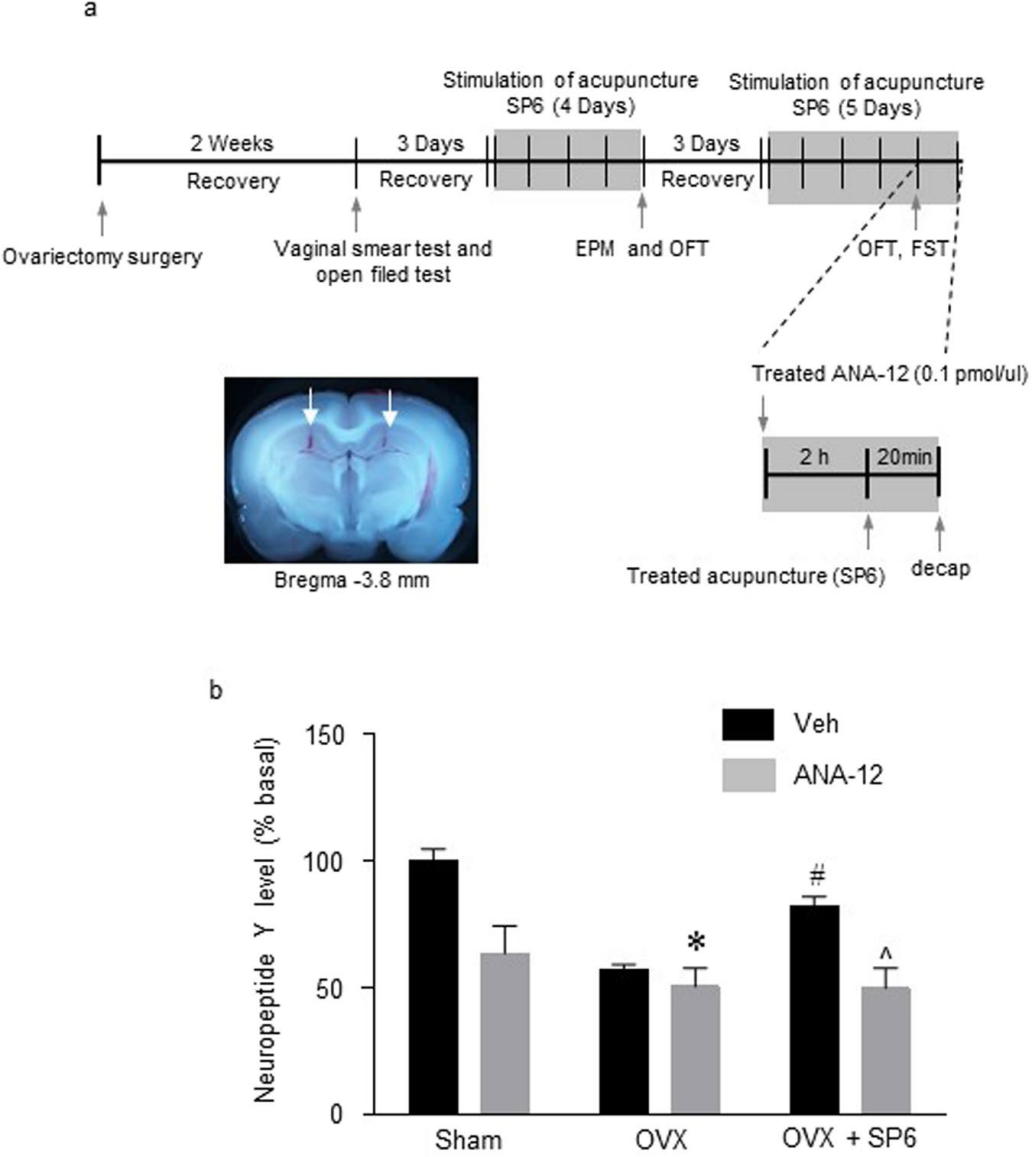
Effects of a BDNF receptor antagonist on the changes in neuropeptide Y levels induced by acupuncture stimulation. (**a**) A schematic illustration showing the construction of the microinjection schedule and needle placement in the hippocampus. (**b**) The release of NPY after pretreatment with the TrkB antagonist (ANA-12) before SP6 stimulation in the hippocampus. The data were analyzed using repeated measures ANOVA followed by Tukey’s test. *p < 0.05 vs. Sham + Veh group; ^#^*p* < 0.05 vs. OVX + Veh groups; ^*p* < 0.05 vs. OVX + SP6 + Veh group. Values are expressed as the means ± SEM.

Since MAI stimulation of the SP6 point in the OVX rats increased BDNF, phosphorylated TrkB and NPY, the next experiment was conducted to determine whether phosphorylated TrkB can increase NPY levels as a result of MAI stimulation of that point. The TrkB receptor antagonist ANA-12 (0.01 pmol) was applied before MAI stimulation of the acupuncture point (Fig. 7a). NPY expression was significantly greater in the OVX + Vehicle (Veh) + SP6 group than in the OVX + Veh group. However, NPY expression of the OVX + ANA-12 + SP6 group was not significantly increased in the hippocampus (Interaction F_(2, 16)_ = 8.766; SP6 effect F_(1, 16)_ = 38.25, ANA-12 effect F_(2, 16)_ = 15.59, *df* = 15, n = 4 per group) (Fig. 7b). These findings suggest that MAI stimulation of the SP6 acupuncture point increased NPY levels through BDNF release and phosphorylation of the TrkB receptor.

## Discussion

Our results demonstrated that estrogen deficiency increases immobility time during the FST, decreases total travel distance during the OFT, and decreases open-arm exploration during the EPM. In addition, estrogen-deficient rats showed decreased BDNF and NPY expression in the hippocampus. However, MAI stimulation at SP6 significantly reduced estrogen deficit-induced depression-like behaviors and increased BDNF and NPY. In particular, increased NPY levels are mediated by phosphorylated TrkB receptor activation dependent on BDNF release in the hippocampus. Thus, this study suggests that the effect of MAI stimulation at SP6 might be attributable to changes in a pathway that is modulated by the BDNF-NPY system and relates to depression-like behaviors. Notably, we report the novel finding that MAI stimulation at SP6 relieves depression-like behavior through increased hippocampal BDNF rather than an increase in estrogen.

The ovary is the main source of circulating estrogen that affects the estrous cycle; thus, we used OVX rats as an animal model of menopausal mood disorder. However, this OVX model does not exclude potential effects from local E2 or aromatized testosterone. Two weeks after ovariectomy, we confirmed the success of the surgery in the OVX rats by observing decreased serum E2 levels and no mature exfoliated epithelial cells in vaginal smears. After we confirmed the absence of estrus, we applied MAI stimulation to the acupuncture point at SP6 (Fig. 1b,c).

Loss of estrogen is believed to be the cause of many menopausal symptoms, including depression. According to previous studies, acupressure or stimulation at SP6 improved women’s general health including menopausal depression^46–49^. In various depression studies, stimulation of certain acupuncture points decreased depression-like behaviors in rats^38^. Acupuncture at Neiguan (PC6) has a therapeutic effect on chronic stress-related diseases such as anxiety and depression^50^. In the chronic unpredictable mild stress (CUMS) rat model, electroacupuncture (EA) acts on depression via the hippocampus^51^. The first experiment was conducted to determine whether stimulation of SP6 alleviates depression-like behaviors in OVX rats. Our results demonstrated that MAI stimulation of the acupuncture point at SP6 also reduces depression-like behavior on measures such as the OFT (total distance traveled), EPM (open arms spent time) and FST (immobility time) in the OVX rat model. No significant differences were detected among other depression-like behaviors. Based on the statistically significant differences in the behavioral results, we investigated the mechanism by which MAI stimulation at SP6 reduced depression-like behaviors.

Depression-like behaviors were reduced by estradiol injection in the OVX rat model^36^. In the EA stimulation study, a needle retention time of 40 min significantly increased serum E2 and progesterone^39^. In addition, EA can suppress menopause-induced down-regulation of ovarian E2 production and hypothalamic estrogen receptor protein in menopausal rats^52,53^. For this reason, this study tested whether MAI stimulation at SP6 could restore estrogen deficiency in the hippocampus to reduced depression-like behaviors. According to our results, interestingly, E2 levels in the hippocampus as well as plasma were not restored 20 min after MAI stimulation. In addition, ERβ expression in the hippocampus did not recover. Because of the differences in stimulation time and stimulation type, it is not surprising that our result is different from those of previous studies. In other words, MAI stimulation at SP6 does not, by itself, increase the estrogen level. Our results indicate that the antidepressant-like behavioral effects of MAI stimulation at SP6 may regulate other pathways.

It is known that, even without estrogen hormonal regulation, acupuncture stimulation regulates depression-like behavior by modulating various factors such as neurotrophins and neuropeptides. A recent study found that stimulation of various acupuncture points increased BDNF expression levels. Acupuncture stimulation at PC6 increased BDNF gene and protein expression in the hippocampus and prefrontal cortex in a chronic stress rat model of depression^40^. In addition, acupuncture stimulation at BaegHoe (GV20) may be useful to improve cognitive functioning in numerous neurodegenerative diseases by stimulating cholinergic enzyme activity and regulating BDNF in the brain^41^. In this study, MAI stimulation at SP6 increased the level of mature BDNF and phosphorylated TrkB receptors in the hippocampus. BDNF is initially synthesized as precursor BDNF and is proteolytically processed to become mature BDNF. Although there are many studies showing that BDNF is increased by acupuncture stimulation, no study has separately examined pro- and mature BDNF. Our results showed that only mature BDNF increased in response to MAI treatment.

On the other hand, NPY also ameliorates depression through mechanisms involving synaptic transmission and neuroprotection^34^. A recent study found that stimulation of various acupuncture points increased the expression of NPY. Acupuncture at PC6 reduces chronic corticosteroid-induced depression- and anxiety-like behavior via modulation of NPY expression levels^38^. Shenmen (HT7) stimulation increases NPY expression in the hippocampus of maternally separated rats. In this study, MAI stimulation at SP6 also increased NPY expression in the hippocampus. Our results indicate that the antidepressant-like behavior effects of MAI stimulation at SP6 involved increased mature BDNF expression, activation of phosphorylated TrkB and increased NPY expression in the hippocampus. However, the signal relationship between BDNF and NPY is not established by the preceding results. It is necessary to investigate whether MAI stimulation increases each of these separately or whether NPY is affected by BDNF.

After blockade of the TrkB receptor, NPY expression was not recovered in the MAI-treated OVX rats. This result indicates that MAI-induced recovery of hippocampal NPY expression may depend on phosphorylated TrkB activation in the hippocampus of the OVX rats. Our results plausibly explain the signaling cascade connecting BDNF and NPY expression in the hippocampus in response to MAI stimulation.

**Figure 8:**
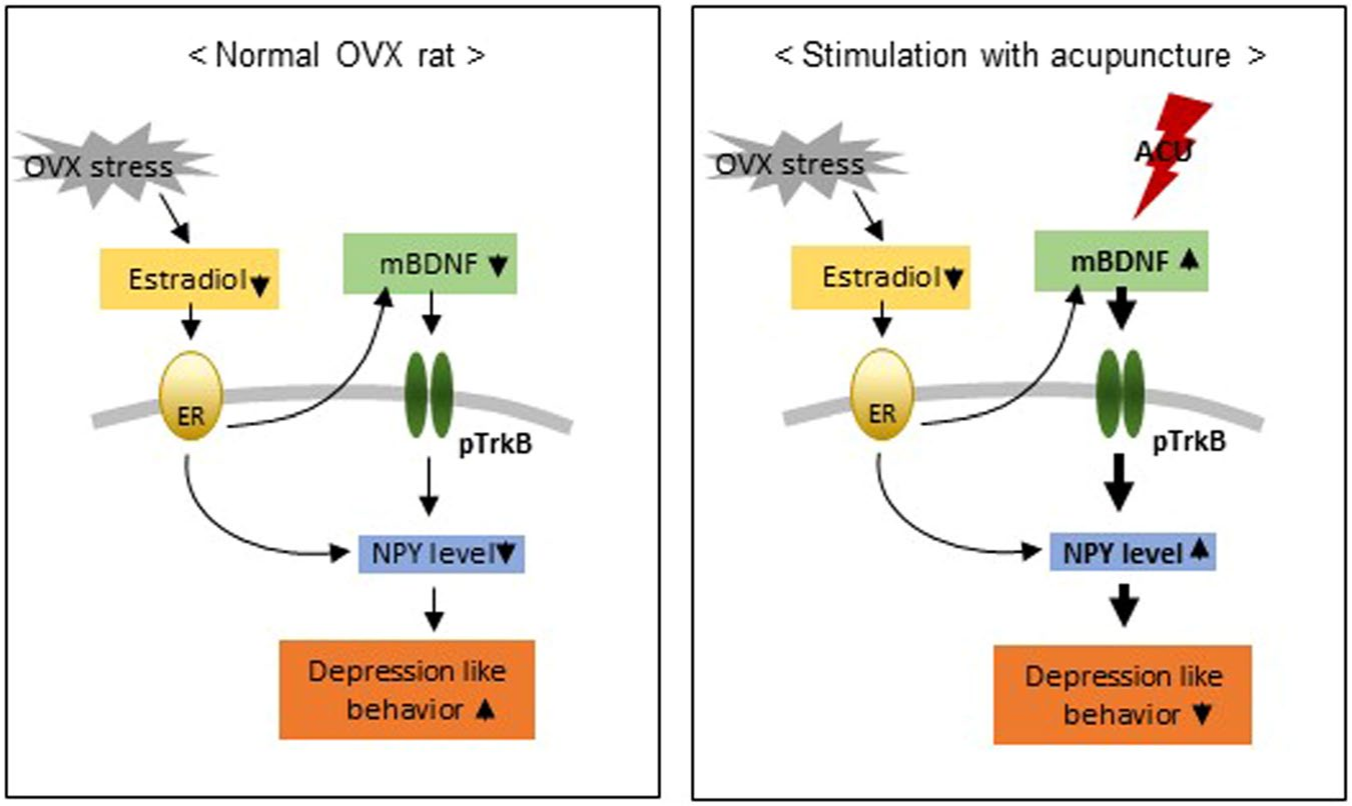
Schematic diagrams illustrating the mechanism of the effects of acupuncture stimulation. The proposed mechanism of BDNF and NPY interactions and the effects of acupuncture.

Although our results do not demonstrate how MAI stimulation increased BDNF expression and TrkB phosphorylation without increasing E2 levels, we can identify a potential underlying mechanism. Acupuncture has been shown to directly promote the extracellular signal-regulated kinase (ERK) signaling pathway. Acupuncture increases ERK1/2 phosphorylation and subsequent cAMP response element binding protein (CREB) phosphorylation in CUMS rats^54,55^. In conclusion, the present study demonstrates that MAI stimulation at SP6 in OVX rats relieves anxiety- and depression-like behaviors, not by directly increasing deficient estrogen levels but by increasing the formation of the BDNF-TrkB binding complex, which subsequently boosts hippocampal NPY levels (Fig. 8). Taken together, our findings suggest that acupuncture is a potentially effective therapeutic alternative for treating patients suffering from postmenopausal symptoms, especially anxiety and depressive disorders.

## Methods

### Animals and groups

Adult female Sprague-Dawley rats (250–300 g) were obtained from Orient Bio (Seongnam, Korea). The rats were allowed to acclimate for a minimum of seven days, housed in pairs in a controlled environment, and maintained on a 12 h/12 h light/dark cycle during all experimental treatments. In addition, temperature and humidity were maintained at 20–23 °C and 45–55%, respectively. Food and water were provided *ad libitum*. On experiment days, the acupuncture treatment and behavioral experiments were conducted in a quiet room to minimize stress to the animals. The experimental protocols for animal usage were reviewed and approved by the Institutional Animal Care and Use Committee of Korea Institute of Oriental Medicine with reference number #17-009 (Daejeon, Korea).

The experiments were designed to investigate the effects of acupuncture on OVX-induced depression-like behavioral responses and associated protein expression. In preliminary studies, we utilized a 2 × 3 group design: Sham, Sham + SP6, Sham + NonAcu, OVX, OVX + SP6 and OVX + NonAcu. No significantly differences were observed among the Sham, Sham + SP6, and Sham + NonAcu groups. Therefore, further experiments were conducted using the fewest groups (Sup. 4). The rats were divided into four groups and subjected to the following treatments: Sham, OVX alone, OVX with bilateral acupuncture at SP6 (located 3 cun directly above the tip of the medial malleolus on the posterior border of the tibia) and OVX with stimulation at a non-acupuncture point (located at the upper part of the left buttock). An MAI was used to administer stimulation once daily for 4 consecutive days. The MAI was developed to mimic the vibrations produced by manual acupuncture stimulation^56^. The MAI was obtained from Daegu Haany University (Daegu, Korea). The acupuncture needles (0.3 × 30 mm, DongBang medical, Gyeongi-do, Korea) were inserted into acupuncture point SP6 and the selected non-acupoint, vibrated with MAI for 30 s, maintained in place up to 1 min after insertion and subsequently withdrawn.

### OVX surgery

The female rats were ovariectomized under inhalation anesthesia (4% isoflurane in oxygen)^57^. An abdominal incision was made through the skin of the flank, and the ovaries were removed. The same surgery was performed on the Sham rats, except that the wound was closed without removing the ovaries^58^. Two weeks after surgery, the difference between the OVX group and Sham group was verified by a vaginal smear test (Fig. 1b).

### Behavioral assessments

Rats were placed in a dark room 30 min before behavioral tests, which were conducted 20 min after MAI stimulation in the sequence of OFT and EPM. The OFT and EPM were run on the same day. After a 3-day recovery period, MAI stimulation was started again (Fig. 1a). The FST was conducted 20 min after last MAI stimulation. All tests took place between 10:00 and 12:00 a.m. with a randomized block in a dimly lit room (±20 lx). After each test, rats were returned to their home cages and then to the holding room once every animal was tested.

#### Forced swim test (FST)

The ovariectomized rats were placed in a cylindrical glass tank (60 cm height and 38 cm width), which was filled with water (24 ± 1 °C) to a depth of 40 cm to prevent the rats from supporting themselves by touching the bottom with their feet. After being forced to swim for 5 min, the rats were removed from the cylinders. The water in the cylinder was replaced after every trial. Five minutes of forced swimming was videotaped from the front of the cylinder. To score several types of behavior, this experiment used a time-sampling technique. The scoring was conducted by a blinded experimenter using a SMART v3.0 tracking system (Panlab, Barcelona, Spain), which determined the immobility time.

#### Open-field test (OFT)

The OFT was performed following standard protocols to measure spontaneous activity in rodents. Briefly, the apparatus, consisting of a black square cage 120 cm × 120 cm × 40 cm in size, was divided into equal 60 cm × 60 cm squares along the floor. The test room was dimly lit. A single rat was placed in the center of the cage, and after 30 s of adaptation, the total travel distance was recorded. Although various depression-like behavioral indicators were also measured, for example, resting time and rearing duration, these were excluded from the results because no significant differences were observed (Sup. 1). Images were captured on a computer with a SMART v3.0 tracking system for 5 min. After each test, the arena was cleaned with a 70% alcohol solution.

#### Elevated plus maze (EPM)

After OFT, the rats were transferred to the EPM, a four-armed platform in the shape of a plus sign. The apparatus was coated with black enamel and was raised 50 cm above the floor. All arms were 10 cm in width, 50 cm in length, and joined in the center to create a 10 cm^2^ center platform. Two opposite arms were closed, whereas the remaining two arms remained open. At the start of the test, the rats were allowed to move freely for 3 min. The video footage of these sessions was scored. The test was scored by a blinded experimenter using a SMART v3.0 tracking system. The distance moved and frequency of entering the closed and open arms were quantified. However, while the closed arm entering time showed a tendency to be changed by MAI stimulation, the difference was not statistically significant (Sup. 1).

### ELISA assay

The E2, mature BDNF, pro-BDNF and NPY levels were detected using chemiluminescence. An E2 ELISA kit (Biovision, Milpitas, CA, USA; catalog no. K7417-100), a mature BDNF ELISA kit (Promega, Wisconsin, USA; catalog no. BEK-2211), a pro-BDNF ELISA kit (Promega; catalog no. BEK- BEK-2217) and a neuropeptide Y ELISA kit (Cusabio Biotech Co., Wuhan, China; catalog no. CSB-EL016037RA) were used to detect the levels of the corresponding substances. The detailed steps were carried out according to the manufacturer’s protocol.

### Western blotting

The rats were subjected to inhalation anesthesia with 4% isoflurane in oxygen and sacrificed 20 min after the final acupuncture treatment. The brains were serially cut in a rodent brain matrix, and the hippocampus was removed. All tissue samples were lysed in RIPA lysis buffer (50 mM Tris pH 7.4, 50 mM NaCl, 0.1% SDS, 1% NP-40, 0.5% DOC, 1 mM EGTA, 1 mM PMSF, 1 mM Na_3_VO_4_, 1 mM Na-F, 1 g/ml leupeptin, 1 g/ml aprotinin). The samples were sonicated for 30 s on ice and incubated for 1 h at 4 °C. The samples were centrifuged again at 13,000 rpm for 30 min at 4 °C to obtain samples free of large debris. The fractionated proteins were used for 10% sodium dodecyl sulfate-polyacrylamide gel (Bio-Rad Laboratories, CA, USA) electrophoresis, and the separated proteins were transferred to a nitrocellulose membrane. The concentration of proteins in the supernatant was determined based on the Bradford method using a Bio-Rad Protein Assay (Bio-Rad Laboratories). The membrane was blocked with blocking buffer containing 5% skim milk in mixture of tris-buffered saline and TWEEN 20 (TBST) and then probed with primary antiserum against ERβ (1:1000 Abcam, Cambridge, MA, USA; catalog no. ab3576), pTrkB (1:1000, Abcam; catalog no. ab109684), TrkB (1:1000, Abcam; catalog no. ab18987) or β-actin (1:2000, Cell Signaling Technology, MA, USA; catalog no. 4967) overnight at 4 °C on a shaker. After 3 washes with TBST for 10 min, the membrane was incubated with the appropriate secondary antiserum (KPL, MD, USA) at a 1:2000 dilution for 2 h at room temperature (RT). Membranes containing immunoreactive protein bands were developed using enhanced chemiluminescence reagents (Thermo Fisher). The protein bands were detected using a Fusion SL4-imaging system (Vilber Lourmat, Eberhardzell, Germany), and quantification of the immunoblotting bands was performed with ImageJ.

### Immunofluorescent staining

To examine BDNF and NPY release, immunofluorescent staining was performed. Hippocampal tissue was fixed in 10% neutral buffered formalin (Sigma-Aldrich, Saint Louis, MO, USA), embedded in paraffin and cut into 10-μm-thick sections. Next, the sections were deparaffinized, hydrated and stained using standard methods. For immunohistochemistry, the hippocampal sections were deparaffinized. Antigen retrieval was performed by heating the tissue in antigen unmasking buffer (Vector Labs, Burlingame, CA) for 10 min in a microwave. Specimens were blocked in 5% blocking solution for 30 min at RT, followed by incubation with primary antibodies at 4 °C overnight. An anti-BDNF rabbit polyclonal antibody (diluted 1:500; Santa Cruz Biotechnology, Santa Cruz, USA; catalog no. sc546) and anti-NPY rabbit polyclonal antibody (diluted 1:500; Cell Signaling Technology; catalog no. 11976) were used as primary antibodies and were diluted in PBS/0.2% Triton-X-100. The secondary antibodies used here were Alexa Fluor 647- conjugated anti-rabbit and Alexa Fluor 488-conjugated anti-rabbit, which corresponded to the species of the primary antibodies (1:1000; Thermo Fisher). The coverslips were then dried and mounted onto slides using Vectashield with DAPI (Vector Laboratories). The microscopy was performed using a fluorescence microscope (BX51; Olympus, Hamburg, Germany). The images were collected and then processed using an image processor (CellSens 1.41, Olympus Software, Tokyo, Japan).

### Intrastriatal infusion

The female rats were anesthetized with 4% isoflurane in oxygen. The skull was exposed, and the head was positioned in a stereotaxic apparatus. A 10-μL Hamilton syringe (Hamilton, Bonaduz AG, Bonaduz, Switzerland) with a stainless needle was used to inject 0.1 pmol/1 μL ANA-12 (Tocris, Bristol, UK, pH 7.4 adjusted with NaOH) or aCSF into the hippocampus with an infusion pump (UMC4; World Precision Instruments, FL, USA) over the course of 2 min. The needle was left in place for another 5 min before removal, and the total procedure lasted 7 min. The coordinates for injection were as follows: anterior/posterior (AP) −3.8 mm, lateral (L) +/−2.0 mm, and dorsal/ventral (DV) −2.5 mm^59^. The correct position of the needle was assessed by brain slide capture (Fig. 7a). The ANA-12 dose and administration method were based on a previous work^60^. Postoperatively, the rats remained in a warm cage until they recovered.

### Statistics

Differences in the number of immunoreactive pixels per measured area, total distance traveled, duration of open-arm and immobility time between groups performed in this study were determined by one-way ANOVA, two-way ANOVA or t-test followed by Tukey’s honest significant difference test using GraphPad Prism 6 (GraphPad Software Inc., San Diego, CA, USA). Data are expressed as the mean ± SEM for each group. A p value < 0.05 was considered statistically significant.

## Electronic supplementary material

Supplementary Information
