# Supplementary material for: An estradiol-independent BDNF-NPY cascade is involved in the antidepressant effect of mechanical acupuncture instruments in ovariectomized rats
Source: Sci Rep. 2018 Apr 11;8:5849. doi: 10.1038/s41598-018-23824-2 (PMC5895789; doi:10.1038/s41598-018-23824-2)
Supplement: Supplementary file 1 — Supplementary Information [file 41598_2018_23824_MOESM1_ESM.docx]

**Scientific reports**

**An estradiol-independent BDNF-NPY cascade is involved in the antidepressant effect of mechanical acupuncture instruments in ovariectomized rats**

Su Yeon Seo^1^, Ji-Young Moon^1^, Suk-Yun Kang^1^, O Sang Kwon^1^, Sunoh Kwon^1^, Se kyun Bang^1^, Soo Phil Kim^1^, Kwang-Ho Choi^1^, Yeonhee Ryu^1^*

^1^Korea Institute of Oriental Medicine 1672 Yuseongdae-ro, Yuseong-gu,

Daejeon, 34054, Korea

*Corresponding author

Tel.: +82 42 868 9484; fax: +82 42 863 9464

E-mail address: [yhryu@kiom.re.kr](mailto:yhryu@kiom.re.kr) (Y.H. R)

**Supplementary 1**

**
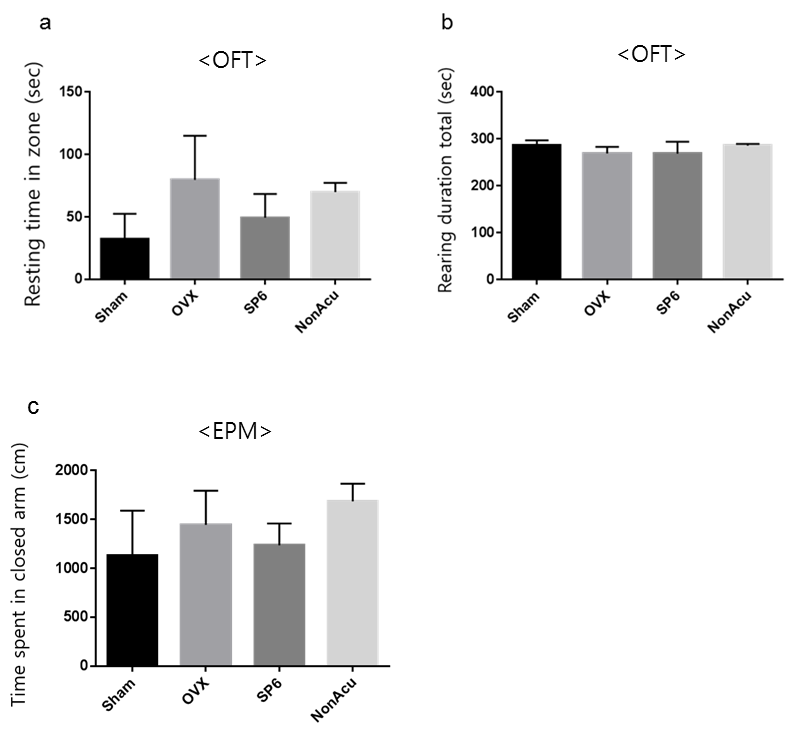
**

**Supplementary 1. Effects of acupuncture stimulation on depression-like behaviors in main figure 2.**

Quantification of (a) resting time and (b) rearing duration total time in the OFT (n = 10 for each group) and the entries into the closed arms in the EPM (n = 7 for each group). The data were analyzed using repeated measures ANOVA followed by Tukey’s test. **p* < 0.05 vs. Sham group; # *p* < 0.05 vs. OVX group. Values are expressed as the means ± SEM.

**Supplementary 2**

**
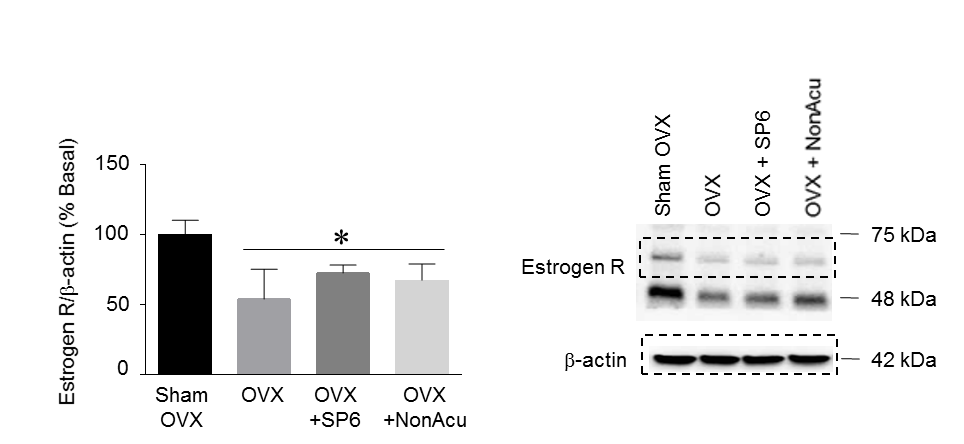
**

**Supplementary 2. Full-length pictures of the blots presented in the main figure 3c.** The data were analyzed using repeated measures ANOVA followed by Tukey’s test. **p* < 0.05 vs. Sham group. Values are expressed as the means ± SEM.

**Supplementary 3**
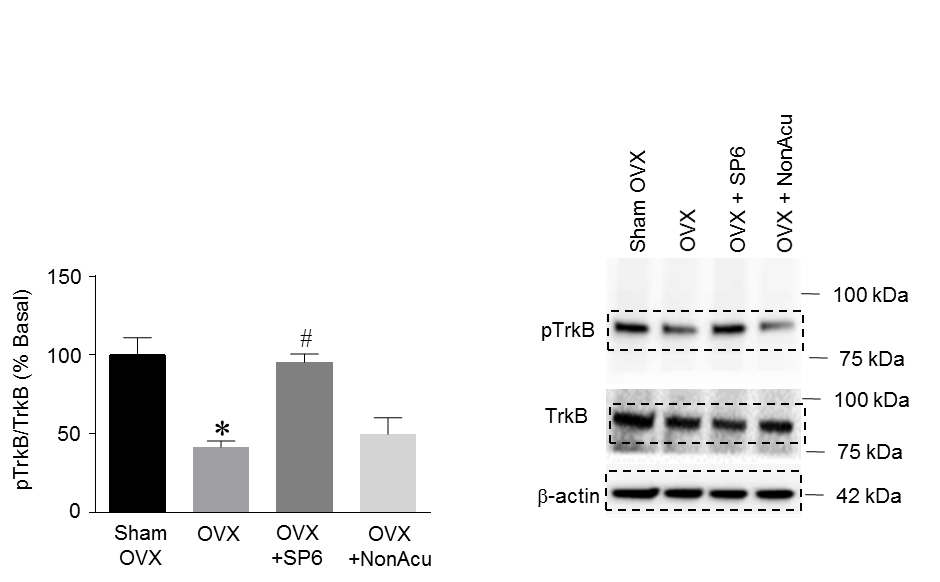


**Supplementary 3. Full-length pictures of the blots presented in the main figure 5.** The data were analyzed using repeated measures ANOVA followed by Tukey’s test. **p* < 0.05 vs. Sham group; # *p* < 0.05 vs. OVX group. Values are expressed as the means ± SEM.

**Supplementary 4**

**
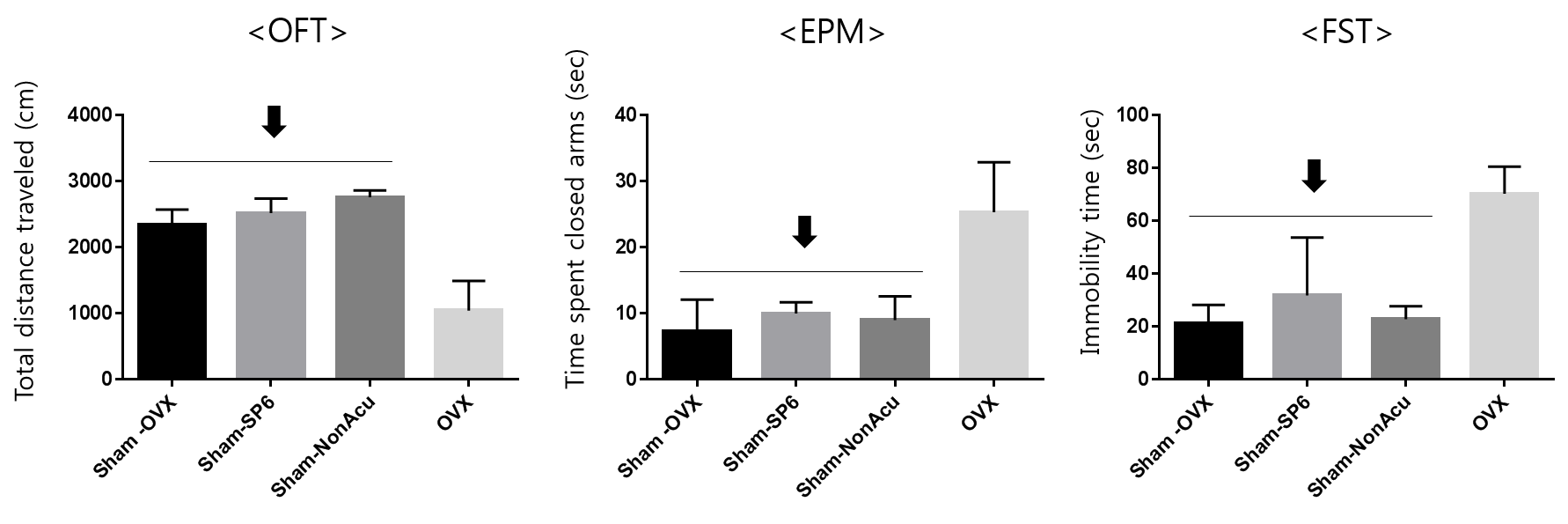
**

**Supplementary 4. The groups (Sham, Sham + SP6, Sham + NonAcu, OVX) of behavioral experiments in main figure 2.**
